# Neurophysiological Effects of Trait Empathy in Music Listening

**DOI:** 10.3389/fnbeh.2018.00066

**Published:** 2018-04-06

**Authors:** Zachary Wallmark, Choi Deblieck, Marco Iacoboni

**Affiliations:** ^1^Meadows School of the Arts, Southern Methodist University, Dallas, TX, United States; ^2^Ahmanson-Lovelace Brain Mapping Center, University of California, Los Angeles, Los Angeles, CA, United States; ^3^Academic Center for ECT and Neuromodulation, University Psychiatric Center, University of Leuven, Leuven, Belgium; ^4^Department of Psychiatry and Biobehavioral Sciences, Semel Institute for Neuroscience and Human Behavior, David Geffen School of Medicine, University of California, Los Angeles, Los Angeles, CA, United States

**Keywords:** empathy, music cognition, social neuroscience, affective neuroscience, fMRI

## Abstract

The social cognitive basis of music processing has long been noted, and recent research has shown that trait empathy is linked to musical preferences and listening style. Does empathy modulate neural responses to musical sounds? We designed two functional magnetic resonance imaging (fMRI) experiments to address this question. In Experiment 1, subjects listened to brief isolated musical timbres while being scanned. In Experiment 2, subjects listened to excerpts of music in four conditions (familiar liked (FL)/disliked and unfamiliar liked (UL)/disliked). For both types of musical stimuli, emotional and cognitive forms of trait empathy modulated activity in sensorimotor and cognitive areas: in the first experiment, empathy was primarily correlated with activity in supplementary motor area (SMA), inferior frontal gyrus (IFG) and insula; in Experiment 2, empathy was mainly correlated with activity in prefrontal, temporo-parietal and reward areas. Taken together, these findings reveal the interactions between bottom-up and top-down mechanisms of empathy in response to musical sounds, in line with recent findings from other cognitive domains.

## Introduction

Music is a portal into the interior lives of others. By disclosing the affective and cognitive states of actual or imagined human actors, musical engagement can function as a mediated form of social encounter, even when listening by ourselves. It is commonplace for us to imagine music as a kind of virtual “persona,” with intentions and emotions of its own (Watt and Ash, 1998; Levinson, 2006): we resonate with certain songs just as we would with other people, while we struggle to identify with other music. Arguing from an evolutionary perspective, it has been proposed that the efficacy of music as a technology of social affiliation and bonding may have contributed to its adaptive value (Cross, 2001; Huron, 2001). As Leman (2007) indicates: “Music can be conceived as a virtual social agent … listening to music can be seen as a socializing activity in the sense that it may train the listener’s self in social attuning and empathic relationships.” In short, musical experience and empathy are psychological neighbors.

The concept of empathy has generated sustained interest in recent years among researchers seeking to better account for the social and affective valence of musical experience (for recent reviews see Clarke et al., 2015; Miu and Vuoskoski, 2017); it is also a popular topic of research in social neuroscience (Decety and Ickes, 2009; Coplan and Goldie, 2011). However, the precise neurophysiological relationship between music processing and empathy remains unexplored. Individual differences in trait empathy modulate how we process social stimuli—does empathy modulate music processing as well? If we consider music through a social-psychological lens (North and Hargreaves, 2008; Livingstone and Thompson, 2009; Aucouturier and Canonne, 2017), it is plausible that individuals with a greater dispositional capacity to empathize with others might also respond to music-as-social-stimulus differently on a neurophysiological level by preferentially engaging brain networks previously found to be involved in trait empathy (Preston and de Waal, 2002; Decety and Lamm, 2006; Singer and Lamm, 2009). In this article, we test this hypothesis in two experiments using functional magnetic resonance imaging (fMRI). In Experiment 1, we explore the neural correlates of trait empathy (as measured using the Interpersonal Reactivity Index) as participants listened to isolated instrument and vocal tones. In Experiment 2, excerpts of music in four conditions (familiar liked/disliked, unfamiliar liked/disliked) were used as stimuli, allowing us to examine correlations of neural activity with trait empathy in naturalistic listening contexts.

### Measuring Trait Empathy

Trait empathy refers to the capacity for empathic reactions as a stable feature of personality. Individual differences in trait empathy have been shown to correlate with prosocial behavior (Litvack-Miller et al., 1997; Balconi and Canavesio, 2013) and situational, “state” empathic reactions to others (Bufalari et al., 2007; Avenanti et al., 2009). Trait empathy is commonly divided into two components: *emotional empathy* is the often unconscious tendency to share the emotions of others, while *cognitive empathy* is the ability to consciously detect and understand the internal states of others (Goldman, 2011).

There are a number of scales to measure individual differences in trait empathy currently in use, including the Toronto Empathy Questionnaire (TEQ), Balanced Emotional Empathy Scale (BEES), Empathy Quotient (EQ), Questionnaire of Cognitive and Affective Empathy (QCAE) and Interpersonal Reactivity Index (IRI). Here we use the IRI (Davis, 1980, 1996), which is the oldest and most widely validated of these scales and frequently used in neurophysiological studies of empathy (Jackson et al., 2005; Gazzola et al., 2006; Pfeifer et al., 2008; Avenanti et al., 2009; Christov-Moore and Iacoboni, 2016). The IRI consists of 28 statements evaluated on a 5-point Likert scale (from “does not describe me well” to “describes me very well”). It is subdivided into four subscales meant to tap different dimensions of self-reported emotional and cognitive empathy. Emotional empathy is represented by two subscales: the *empathic concern* scale (hereafter EC) assesses trait-level “other-oriented” sympathy towards misfortunate others, and the *personal distress* scale (PD) measures “self-oriented” anxiety and distress towards misfortunate others. The two cognitive empathy subscales consist of *perspective taking* (PT), or the tendency to see oneself from another’s perspective, and *fantasy* (FS), the tendency to imaginatively project oneself into the situations of fictional characters.

### Music and Empathy

Theories of empathy have long resonated with the arts. The father of the modern concept of empathy, philosopher Theodor Lipps (1907), originally devised the notion of *Einfühlung* (“feeling into”) in order to explain aesthetic experience. Contemporary psychological accounts have invoked mirror neurons as a possible substrate supporting Lipps’s “inner imitation” theory of the visual and performing arts (Molnar-Szakacs and Overy, 2006; Freedberg and Gallese, 2007). However, the incorporation of psychological models of empathy in empirical music research is still in its early stages. Empathy remains an ambiguous concept in general (Batson, 2009), but applications to music can appear doubly vexed. In an influential formulation, Eisenberg et al. (1991) define empathy as, “an emotional response that stems from another’s emotional state or condition and is congruent with the other’s emotional state or condition.” Aspects of this definition, though, might seem incongruous when applied to music, which is inanimate and not capable of possessing an emotional “state” (Davies, 2011). To connect music processing to trait empathy, therefore, it is first necessary to determine the extent to which music comprises a social stimulus. Who or what do we *empathize with* when listening to music?

Scherer and Zentner (2001) proposed that empathy toward music is often achieved via identification and sympathy with the lived experiences and expressive intentions of composers and performers. Corroborating this view, in a large web-based experiment Egermann and McAdams (2013) found that “empathy for the musician” moderated between recognized and induced emotions in music: the greater the empathy, the more likely an individual was to exhibit a strong affective response when listening. In a related study, Wöllner (2012) presented participants with video of a string quartet performance in three conditions—audio/visual, visual only, and audio only—and reported a significant correlation between trait empathy measures and perceived expressiveness in both visual conditions (music-only condition was non-significant), leading him to conclude: “since music is the audible outcome of actions, empathic responses to the performer’s movements may enhance the enjoyment of music.” Similarly, Taruffi et al. (2017) found correlations between the EC and FS scales of the IRI and accuracy in emotion recognition relative to musicians’ self-reported expressive encodings in an audio-only task.

A music-specific manifestation of trait empathy was proposed by Kreutz et al. (2008), who defined “music empathizing” as a cognitive style of processing music that privileges emotional recognition and experience over the tendency to analyze and predict the rules of musical structure (or, “music systematizing”). Garrido and Schubert (2011) compared this “music empathy” scale alongside the IRI-EC subscale in a study exploring individual differences in preference for sad music. They found that people who tend towards music empathizing are more likely to enjoy sad music; however, high trait empathy was not significantly correlated with enjoyment of sad music. This would seem to suggest that the music empathizing cognitive style differs from general trait empathy. A number of other studies have investigated the relationship between trait empathy and enjoyment of sad music using the IRI. In a series of experiments, Vuoskoski and Eerola (2011), Vuoskoski et al. (2012) and Eerola et al. (2016) reported statistically significant correlations between EC and FS subscales and self-reported liking for sad and tender music. Similarly, Kawakami and Katahira (2015) found that FS and PT were associated with preference for and intensity of emotional reactions to sad music among children.

There is evidence that musical affect is often achieved through mechanisms of emotional empathy (Juslin and Västfjäll, 2008). According to this theory, composers and performers encode affective gestures into the musical signal, and listeners decode that signal by way of mimetic, mirroring processes; musical expression is conveyed transparently as affective bodily motions are internally reenacted in the listening process (Overy and Molnar-Szakacs, 2009). Schubert (2017), in his Common Coding Model of Prosocial Behavior Processing, suggests that musical and social processing draw upon shared neural resources: music, in this account, is a social stimulus capable of recruiting empathy systems, including the core cingulate-paracingulate-supplementary motor area (SMA)-insula network (Fan et al., 2011), along with possible sensorimotor, paralimbic and limbic representations. The cognitive empathy component, which can be minimal, is involved primarily in detecting the aesthetic context of listening, enabling the listener to consciously bracket the experience apart from the purely social. This model may help account for the perceived “virtuality” of musical experience, whereby music is commonly heard as manifesting the presence of an imagined other (Watt and Ash, 1998; Levinson, 2006).

In sum, trait empathy appears to modulate self-reported affective reactions to music. There is also peripheral psychophysiological evidence that primed situational empathy may increase emotional reactivity to music (Miu and Balteş, 2012). Following Schubert (2017), it is plausible that such a relationship is supported by shared social cognitive mechanisms that enable us to process music as a social stimulus; however, this hypothesis has not yet been explicitly tested at the neurophysiological level.

### Neural Correlates of Trait Empathy

Corroborating the bipartite structure of trait empathy that appears in many behavioral models of empathy, two interrelated but distinct neural “routes” to empathy have been proposed (Goldman, 2011), one associated with emotional contagion and the other with cognitive perspective taking. Emotional empathy is conceived as a bottom-up process that enables “feeling with someone else” through perception-action coupling of affective cues (Preston and de Waal, 2002; Goldman, 2006). Such simulation or “mirroring” models maintain that empathy is subserved by the activation of similar sensorimotor, paralimbic and limbic representations both when one observes another and experiences the same action and emotional state oneself (Gallese, 2003; Iacoboni, 2009). This proposed mechanism is generally considered to be pre-reflective and phylogenetically ancient; it has also been linked behaviorally to emotional contagion, or the propensity to “catch” others’ feeling states and unconsciously co-experience them (Hatfield et al., 1994). For example, several imaging studies have found evidence for shared representation of observed/experienced pain in anterior cingulate and anterior insula (Singer et al., 2004; Decety and Lamm, 2006; de Vignemont and Singer, 2006), as well as somatosensory cortex (Bufalari et al., 2007). Similarly, disgust for smells and tastes has been shown to recruit the insula during both perception and action (Wicker et al., 2003; Jabbi et al., 2007), and insula has been proposed as a relay between a sensorimotor fronto-parietal circuit with mirror properties and the amygdala in observation and imitation of emotional facial expressions (Carr et al., 2003). There is also evidence that insula functions similarly in music-induced emotions (Molnar-Szakacs and Overy, 2006; Trost et al., 2015), particularly involving negative valence (Wallmark et al., 2018).

In contrast to emotional empathy, trait cognitive empathy has been conceived as a deliberative tendency to engage in top-down, imaginative transpositions of the self into the “other’s shoes,” with concomitant reliance upon areas of the brain associated with theory of mind (Saxe and Kanwisher, 2003; Goldman, 2006), executive control (Christov-Moore and Iacoboni, 2016), and contextual appraisal (de Vignemont and Singer, 2006), including medial, ventral and orbital parts of the prefrontal cortex (PFC; Chakrabarti et al., 2006; Banissy et al., 2012); anterior cingulate (Singer and Lamm, 2009); somatomotor areas (Gazzola et al., 2006); temporoparietal junction (TPJ; Lamm et al., 2011); and precuneus/posterior cingulate (Chakrabarti et al., 2006). As implied in the functional overlap between certain emotional and cognitive empathy circuits, some have argued that the two routes are neither hierarchical nor mutually exclusive (Decety and Lamm, 2006): cognitive perspective taking is premised upon emotional empathy, though it may, in turn, exert top-down control over contagion circuits, modifying emotional reactivity in light of contextual cues and more complex social appraisals (Christov-Moore and Iacoboni, 2016; Christov-Moore et al., 2017b).

Brain studies have converged upon the importance of the human mirror neuron system in action understanding, imitation and empathy (Iacoboni, 2009), and has been demonstrated in multiple sensorimotor domains, including the perception of action sounds (for a review see Aglioti and Pazzaglia, 2010). Mirror properties were initially reported in the inferior frontal gyrus (IFG) and the inferior parietal lobule (IPL; Iacoboni et al., 1999; Shamay-Tsoory, 2010); consistent with simulation theories of trait empathy, moreover, activity in these and other sensorimotor mirror circuits has been found to correlate with IRI scales in a variety of experimental tasks, including viewing emotional facial expressions (all IRI scales; Pfeifer et al., 2008); video of grasping actions (EC and FS; Kaplan and Iacoboni, 2006); and video of hands injected with a needle (PT and PD; Bufalari et al., 2007; Avenanti et al., 2009). That is, high empathy people tend to exhibit greater activation in mirror regions during the observation of others. Simulation mechanisms also appear to underpin prosocial decision-making (Christov-Moore and Iacoboni, 2016; Christov-Moore et al., 2017b). Implication of inferior frontal and inferior parietal mirror neuron areas is not a universal finding in the empathy literature, and some have suggested that it may reflect specific socially relevant tasks or stimulus types, not empathy in and of itself (Fan et al., 2011). However, evidence for mirror properties in single cells of the primate brain now exists in medial frontal and medial temporal cortex (Mukamel et al., 2010), dorsal premotor and primary motor cortex (Tkach et al., 2007), lateral intraparietal area (Shepherd et al., 2009), and ventral intraparietal area (Ishida et al., 2009). This means that in brain imaging data the activity of multiple brain areas may potentially be driven by cells with mirror properties.

In addition to studies using visual tasks, auditory studies have revealed correlations between mirror neuron activity and trait empathy. Gazzola et al. (2006), for instance, reported increased premotor and somatosensory activity associated with PT during a manual action sound listening task. A similar link was observed between IFG and PD scores while participants listened to emotional speech prosody (Aziz-Zadeh et al., 2010). To date, however, no studies have investigated whether individual differences in empathy modulate processing of more socially complex auditory stimuli, such as music.

### Study Aim

To investigate the neural substrates underlying the relationship between trait empathy and music, we carried out two experiments using fMRI. In Experiment 1, we focused on a single low-level attribute of musical sound—*timbre*, or “tone color”—to investigate the effects of empathy on how listeners process isolated vocal and instrumental sounds outside of musical context. We tested two main hypotheses: First, we anticipated that trait empathy (measured with the IRI) would be correlated with increased recruitment of empathy circuits even when listening to brief isolated sounds out of musical context (Gazzola et al., 2006). Second, following an embodied cognitive view of timbre perception (Wallmark et al., 2018), we hypothesized that subjectively and acoustically “noisy” timbral qualities would preferentially engage the emotional empathy system among higher empathy listeners. Abrasive, noisy acoustic features in human and many non-human mammal vocalizations are often signs of distress, pain, or aggression (Tsai et al., 2010): such state cues may elicit heighted responses among people with higher levels of trait EC.

To explore the relationship between trait empathy and music processing, in Experiment 2 participants passively listened to excerpts of self-selected and experimenter-selected “liked” and “disliked” music in familiar and unfamiliar conditions while being scanned. Musical preference and familiarity have been shown to modulate neural response (Blood et al., 1999; Pereira et al., 2011). Extending previous research on the neural mechanisms of empathy, we predicted that music processing would involve circuitry shared with empathic response in non-musical contexts (Schubert, 2017). Unlike Experiment 1, we had no *a priori* hypotheses regarding modulatory effects of empathy specific to each of the four music conditions. However, we predicted in both experiments that emotional empathy scales (EC and PD) would be associated with regions of the emotional empathy system in music listening, including sensorimotor, paralimbic and limbic areas, while cognitive empathy scales (PT and FS) would primarily be correlated with activity in prefrontal areas implicated in previous cognitive empathy studies (Singer and Lamm, 2009; imaging data for both experiments are available online: see Supplementary Material S1 Dataset and S2 Dataset in the online supporting information for NIFTI files of all contrasts reported here).

## Experiment 1

### Methods

#### Subjects

Fifteen UCLA undergraduate students were recruited for the study (eight female; 18–20 years old, *M* age = 19.1, *SD* = 0.72). All were non-music majors; self-reported years of musical training ranged from no experience to 10 years (*M* = 3.27, *SD* = 1.44). Subjects were ethnically diverse (six white, four east Asian, three south Asian, two black), right-handed, normal or corrected-to-normal vision, and had no history of neuropsychiatric disorder. All were paid $25 for their participation. This study was carried out in accordance with the recommendations of the UCLA Office of the Human Research Protection Program with written informed consent from all subjects. All subjects gave written informed consent in accordance with the Declaration of Helsinki. No vulnerable populations were involved as subjects in this research. The protocol was approved following expedited review by the UCLA Institutional Review Board in March 2012. The protocol expired in March 2014 following all data gathering and a 1-year renewal.

#### Stimuli

**Figure 1:**
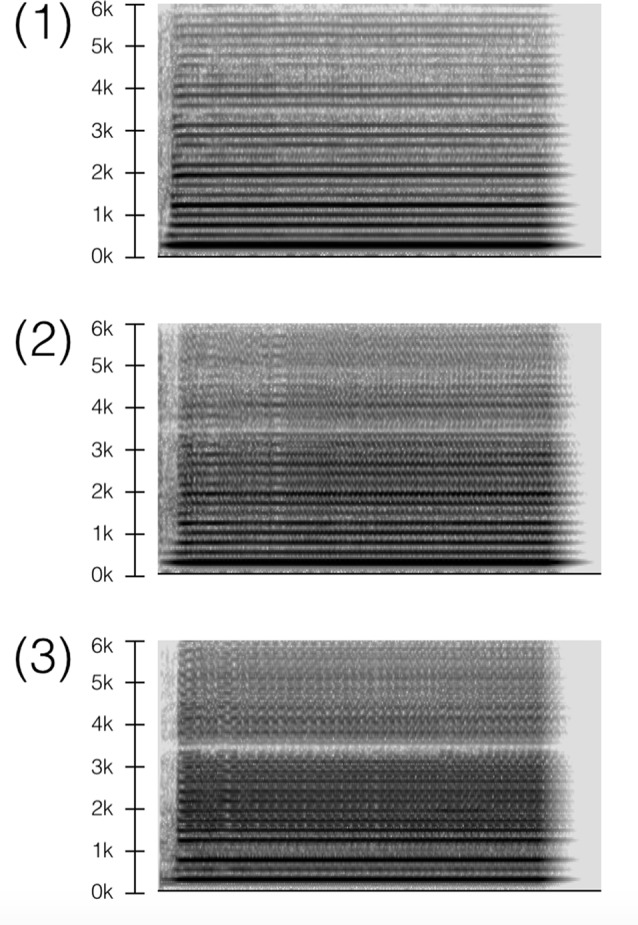
Saxophone stimuli in three conditions: (1) normal, (2) noisy #1, (3) noisy #2. *Spectrograph settings*: size = 1024 with Hanning window, 50% overlap.

We recorded twelve approximately 2-s stimuli (1.8–2.1 s): three electric guitar, three tenor saxophone, three *shakuhachi* (Japanese bamboo flute), and three female vocals. For each sound generator, signals were divided into “normal” and “noisy” versions: (1) normal condition; (2) noisy condition #1; and (3) noisy condition #2. For example, the normal saxophone condition (1) consisted of a regular tone, while noisy conditions (2–3) were growled and overblown to create distortion, as shown in the spectrographs of Figure 1. Noisy signals were characterized acoustically by elevated inharmonicity, spectral centroid, spectral flatness, zero-cross rate, and auditory roughness. Although stimuli were conceived ordinally (normal, medium-noise, high-noise), behavioral and neural evidence suggest that they are perceived dichotomously (i.e., as either not noisy or noisy), as reported in Wallmark et al. (2018). All signals were the same pitch (233 Hz, B♭3) and were manually equalized for loudness. Stimuli were identical to those used in the other study: for complete details, see Wallmark et al. (2018; see Supplementary Material S1 Stimuli).

#### Behavioral Procedure

Fourteen of the fifteen subjects completed the IRI (Davis, 1980) following the scan (one subject did not complete the IRI due to scheduling conflicts). Additionally, in order to evaluate the effect of noisiness levels on valence, subjects rated the stimuli on three covarying perceptual scales using a 0–100 bipolar semantic differential rating scale (Wallmark et al., 2018): (1) *bodily exertion* required to produce each sound (“low exertion-high exertion”), which is generally correlated with acoustic noise in vocal sound production (Tsai et al., 2010); (2) *negative valence* (“like-dislike”); and (3) perceived noisiness (“not noisy-noisy”). Only 10 of the subjects were able to complete this ratings task, also due to scheduling.

#### *MRI* Procedure

Subjects (*N* = 15) listened to the randomized stimuli while being scanned. A sound check prior to the functional scan (conducted with earplugs inserted and the scanner running) allowed subjects to adjust the headphone volume to a subjectively determined comfortable listening level. Participants were then instructed to relax and keep their heads still while listening to the stimuli, and to keep their eyes open and their vision trained on a fixation cross presented through magnet-compatible LCD goggles.

We used a block design consisting of an alternation of 15–16 s baseline period of silence with randomized blocks of all 12 stimuli. Each signal was repeated five times in a row with 100 ms of silence between each onset (9.4–10.9 s total per signal in each block). The full block took approximately 135–140 s, and was repeated three times for a total duration of 405–420 s plus final baseline (around 7.25 m).

#### Data Acquisition, Preprocessing and Statistics

Images were acquired on a Siemens 3T Trio MRI scanner. Functional runs employed a continuous scanning protocol comprised 231 T2-weighted echoplanar images (EPIs; repetition time (TR) 2000 ms; echo time (TE) 28 ms; flip angle = 90°; 34 slices; slice thickness 4 mm; matrix 64 × 64; FOV 192 mm) sensitive to blood oxygenation-dependent (BOLD) contrast. To enable T1 equilibrium the first two volumes of each functional scan were automatically discarded before data collection commenced. Additionally, two sets of structural images were acquired for registration of functional data: a T2-weighted matched-bandwidth high-resolution scan with the same slice prescription as the EPI (TR 5000 ms; TE 34 ms; flip angle = 90°; 34 slices; slice thickness 4 mm; matrix 128 × 128; FOV 192 mm); and a T1 weighted magnetization prepared rapid-acquisition gradient echo image (MPRAGE; TR, 1900 ms; TE 2.26 ms; flip angle = 9°; 176 sagittal slices; slice thickness 1 mm; matrix 256 × 256; FOV 250 mm).

Image preprocessing and data analysis were performed with FSL version 5.0.4. Images were realigned to the middle volume to compensate for any head motion using MCFLIRT (Jenkinson et al., 2002). Volumes were then examined manually for gross motion artifacts that cannot be corrected with simple realignment. When motion artifacts were detected, a nuisance regressor for each affected volume was included in the general linear model (GLM). One run for one subject was excluded for excessive motion (more than 10% volumes exhibiting motion artifacts). Data were temporally filtered with a high-pass filter cutoff of 100 s and spatially smoothed with a 8 mm full width half maximum Gaussian kernel in three dimensions.

Statistical analyses were performed at the single-subject level using a GLM with fMRI Expert Analysis Tool (FEAT, version 6.00). Contrasts included the following: (1) all timbres (task) > baseline; (2) each of the 12 individual stimuli > baseline; (3) intra-instrument comparisons (e.g., Guitar 3 > Guitar 1); (4) inter-timbre comparisons (e.g., all condition 3 > all condition 1); and (5) each instrument > others (e.g., voice > others). Additionally, conditions 1 and 2 were combined for normal > noisy and noisy > normal comparisons. First-level contrast estimates were computed for each run and then registered to standard space (MNI) in three stages. The middle volume of each run of individual EPI data was registered first to the co-planar matched-bandwidth high-resolution T2-weighted image. Following this, the co-planar volume was registered to the T1-weighted MPRAGE. Both of these steps were carried out using FLIRT (affine transformations: EPI to co-planar, *df* = 6; co-planar to MPRAGE, *df* = 6; Jenkinson et al., 2002). Registration of the MPRAGE to MNI space (FSL’s MNI Avg152, T1 2 × 2 × 2 mm) was carried out with FLIRT (affine transformation, *df* = 12). Contrast estimates for each subject were then computed treating each of the three runs as a fixed effect.

Next, group-level analysis was carried out using FSL FLAME stage 1 and 2 (Beckmann et al., 2003). All images were thresholded at *Z* > 2.3, *p* < 0.01, corrected for multiple comparisons using cluster-based Gaussian random field theory controlling family-wise error across the whole brain at *p* < 0.05 (Friston et al., 1994; Forman et al., 1995). In addition to basic group-level contrasts, behavioral ratings were added as continuous covariates to assess the neural correlates of the four IRI scales. Due to moderate (though non-significant) correlations between some of the scales, each of the four IRI covariates were entered into separate second-level analyses. Finally, to isolate regions of covarying activation that correspond to the task, the region of activation from the task > baseline contrast was used to mask the subsequent contrasts so only regions that were also task positive by that criterion showed.

### Behavioral Results

Normality of the distribution of IRI and perceptual data was confirmed (Shapiro-Wilk); to correct for violations in the perceptual dataset, 5 of the 36 variables were transformed using an inverse-normal procedure (Templeton, 2011). Scores for the IRI subscales were then compared using repeated-measures analysis of variance (ANOVA). The test revealed a significant difference between the four scales, *F*_(3,39)_ = 18.28, *p* < 0.0001, $\eta_{\text{p}}^{2}$ = 0.58; *post hoc* testing (Bonferroni) found that mean PD scores were significantly lower than the other three scales. However, subscales were only modestly reliable (*M* Cronbach’s *α* = 0.54). The four scales were not significantly correlated with one another.

To verify whether there was a reliable difference in valence ratings between normal and noisy stimuli, we performed another repeated-measures ANOVA on perceptual ratings data (3 × 4 × 2 design with three perceptual conditions, four sound generators and binary timbral noisiness levels (normal condition 1 and noisy conditions 2 + 3/2)). As expected, no significant main effects of the perceptual scales were revealed (bodily exertion, negative valence, noisiness), *F*_(2,12)_ = 0.6, *p* = 0.94, $\eta_{\text{p}}^{2}$ = 0.01, indicating that all three tapped a similar affective structure (for this reason, only valence was included in subsequent analyses). The main effect of sound generator was significant, *F*_(3,18)_ = 3.34, *p* = 0.04, $\eta_{\text{p}}^{2}$ = 0.34; timbral noisiness also had a large effect on ratings, *F*_(2,12)_ = 7.51, *p* < 0.01, $\eta_{\text{p}}^{2}$ = 0.56, with “noisier” timbres rated significantly lower than the normal condition. Two-way interactions between perceptual categories*sound generator and sound generator*timbral noisiness were likewise significant (*p* < 0.05), and appear to have been driven by the electric guitar and the female voice, which did not differ in bodily exertion and noisiness means but crossed substantially in negative valence and timbral noisiness (voice was perceived as being significantly noisier and more negatively valenced than guitar).

Since many studies have shown a gender difference in IRI scores (Mehrabian et al., 1988; Davis, 1996), we next tested for a behavioral and neural effect of gender on trait empathy. Females showed significantly higher EC scores than males, *t*_(13)_ = 5.44, *p* < 0.0001, Cohen’s *d* = 2.91; no other subscales were significantly different between the sexes. To investigate the possible effect of gender EC differences on imaging results (Derntl et al., 2010), we added sex as a covariate in another second-level analysis. The analysis revealed increased activation of the brain stem among females compared to males in the task > baseline contrast, which is consistent with other studies (Filkowski et al., 2017). However, this result did not survive masking for the task. No other significant differences were found. The same confirmatory analysis was carried out in Experiment 2, which also yielded no significant sex differences aside from EC. Though the sample size for this comparison was small, we concluded for the sake of this study that sex was not a significant neurophysiological factor in music processing.

### Imaging Results

**Figure 2:**
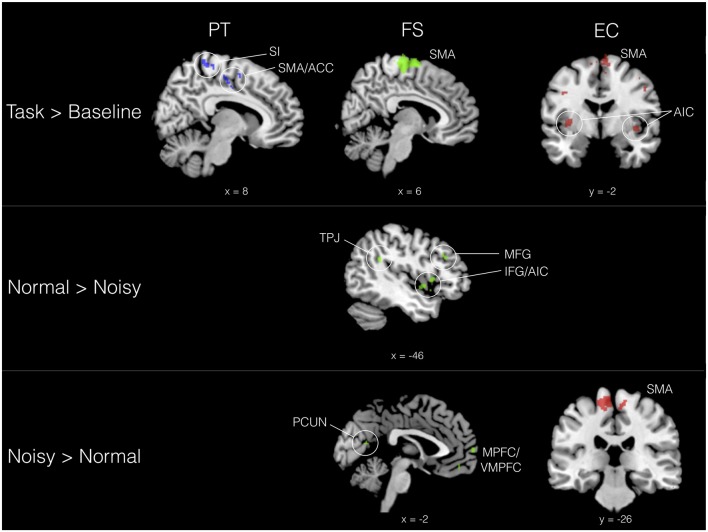
Selected activation sites correlating with trait empathy (IRI subscales) in three contrasts. *Perspective taking* (*PT*) = blue, *fantasy* (FS) = green, *empathic concern* (EC) = red. All contrasts, *Z* > 2.3, *p* < 0.01 (cluster corrected, *p* < 0.05).

We evaluated the effect of trait empathy on the processing of musical timbre in three basic conditions: (1) task > baseline (i.e., sound > silence); (2) positively valenced (normal) > negatively valenced (noisy) timbres; and (3) noisy > normal timbres. Results for these three contrasts are organized according to IRI subscales in Table 1. With trait empathy scores added as covariates to our model, we found that neural responses to the valence of timbre are differentiated by IRI subscale. PT was associated with activation in bilateral sensorimotor areas when listening to aggregated timbres (task > baseline), including SMA and anterior cingulate (ACC), primary motor cortex and primary somatosensory cortex (SI), as shown in Figure 2. FS also involved SMA activation in the task > baseline contrast, in addition to ventrolateral PFC (VLPFC). FS scores were correlated with both directions of the valence contrast: normal timbres modulated activity in left TPJ, inferior/middle frontal gyrus (IFG/MFG) and anterior insula cortex (AIC), while noisy timbres preferentially engaged medial prefrontal (MPFC/VMPFC) and temporal areas, as well as precuneus (PCUN). Both directions of valence modulated activity in IPL.

**Table 1:** Experiment 1 results by interpersonal reactivity index (IRI) subscale.

Contrasts and regions	Cluster extent (voxels)	Maxima			
		MNI coordinates			
		*x*	*y*	*z*	*Z*
***Perspective taking (PT)***					
**Task > Baseline**					
R SMA/anterior cingulate	130	2	−14	54	3.13
L SMA/anterior cingulate	35	−4	−10	64	2.92
L primary somatosensory	30	−36	−24	52	3.39
R primary motor cortex	25	38	−22	48	3.15
R primary somatosensory	24	8	−40	72	3.11
***Fantasy (FS)***					
**Task > Baseline**					
R SMA	487	6	−24	76	4.46
L ventrolateral prefrontal cortex	25	−30	32	−14	3.38
**Normal > Noisy**					
L temporoparietal junction	79	−58	−44	24	3.6
L inferior parietal lobule	40	−56	−42	52	3.22
L inferior frontal gyrus	16	−38	32	2	3.02
L IFG/pars opercularis	15	−46	14	2	2.7
L anterior insula	15	−44	4	−2	2.83
L middle frontal gyrus	12	−44	26	30	2.81
**Noisy > Normal**					
L temporal pole	144	−52	10	−28	3.55
L medial prefrontal cortex	70	−14	68	18	3.54
L inferior parietal lobule	50	−42	−64	28	3.43
R precuneus/posterior cingulate	13	2	−62	16	3.18
***Empathic concern (EC)***					
**Task > Baseline**					
R cerebellum	458	4	−54	−6	4.03
L superior temporal gyrus	327	−34	−32	12	4.23
R SMA	195	10	−14	50	3.54
L superior temporal gyrus	179	40	−34	10	4.41
L temporoparietal junction	147	−58	−42	16	4.86
R anterior insula	92	42	−2	−4	3.25
L cerebellum	92	−24	−66	−46	3.39
L secondary somatosensory cortex	86	−52	−22	20	5.08
L anterior insula	79	−36	0	0	3.55
R primary somatosensory cortex	56	42	−22	44	3.27
L inferior frontal gyrus	50	−50	14	−2	3.32
R occipital fusiform gyrus	49	30	−62	−16	3.16
**Noisy > Normal**					
L SMA	242	−8	−20	66	3.52
R SMA	130	8	−22	62	3.46

*N = 14. Significant voxels were obtained at a threshold of Z > 2.3, p < 0.01 (cluster-corrected, p < 0.05). Brain region labels for all MNI coordinates are based on the Juelich Histological Atlas (Mazziotta et al., 2001). Contrast activations ordered from top to bottom by cluster extent (most to least); brain regions and coordinates listed are derived from peak voxels within each cluster*.

On the emotional empathy scales, we found that in the task > baseline contrast EC modulated activity in a wide swath of bilateral motor (SMA, IPL, IFG), auditory (STG), and somatosensory (SI and SII) areas, in addition to cerebellum and AIC. Some of this sensorimotor activity corresponds to areas also implicated in PT. EC was also correlated with activation of SMA in the noisy > normal contrast, indicating a motor component in the processing of aversive sounds among listeners with higher emotional empathy. PD was not significantly correlated with BOLD signal change in any of the contrasts.

In sum, Experiment 1 demonstrated that trait empathy is correlated with increased activation of circuitry often associated with emotional contagion, including sensorimotor areas and insula, in the perception of isolated musical timbres. FS and EC also appear to be sensitive to the affective connotations of the stimuli. Timbre is arguably the most basic and quickly processed building block of music (Tervaniemi et al., 1997). Though sufficient to recruit empathy areas, these brief stimuli do not, however, constitute “music” *per se*. In Experiment 2, we turned our focus to more naturalistic stimuli—including excerpts of music selected in advance by participants—in order to explore the effect of trait empathy on the processing of music.

## Experiment 2

### Methods

#### Subjects

Twenty UCLA undergraduates (13 female, seven males; 18–20 years old, *M* age = 19.1, *SD* = 0.72) with a range of musical backgrounds were recruited (all non-music-majors; *M* years musical training = 5, *SD* = 3.78). Subjects were ethnically diverse (seven white, five east Asian, four south Asian, two Hispanic, two black), right-handed, normal or corrected-to-normal vision, and had no history of neuropsychiatric disorder. Ten of the subjects also participated in Experiment 1. To ensure that only individuals with strong musical preferences enrolled in the study, we specified in recruitment materials that interested individuals must regularly experience “intense positive and negative emotions when listening to music.” Subjects were paid $50 for their participation. The experiment was approved by the UCLA IRB.

#### Stimuli

Stimuli consisted of sixteen 16-s excerpts of recorded music, half of which were individually selected in pre-scan meetings with each participant. Because musical preference (i.e., liking or disliking) and familiarity have been shown to modulate neural response (Blood et al., 1999; Pereira et al., 2011), we decided to subdivide stimuli into four categories: *familiar liked* (FL), *familiar disliked* (FD), *unfamiliar liked* (UL) and *unfamiliar disliked* (UD). For FL excerpts, subjects brought us four songs they “love” and, for FD, four songs they “hate.” During the meeting and over follow-up communications with subjects, we collaboratively defined the “best” (or “worst”) part of each song for use in the scanner, which typically corresponded to the chorus, the beginning of the first verse, or the introduction. Prior to the scan, all excerpts were approved by the subjects as an accurate reflection of their musical “loves” and “hates.”

The other eight stimuli were selected by the researchers, in consultation with a popular music scholar at UCLA, to match the two categories of self-selected music with UL and UD excerpts. Selections were based on three general criteria: (1) they should roughly match the stylistic and generic features of the familiar songs; (2) they should take into account additional comments relating to musical tastes and affective orientations made by subjects during the meeting; and (3) they should be relatively obscure to typical undergraduate non-music majors, so that the in-scanner hearing represents subjects’ first exposure to the song (for a complete list of stimuli used in the experiment, see Supplementary Material S2 Table 1).

All audio files were trimmed to representative 16-s excerpts with 500-ms amplitude ramps on either end of the signal. Loudness was equalized manually. Control conditions were 16 s of silence (eight times during the MRI run) and a 16-s clip of pink noise (eight times).

#### Procedure

The MRI procedure was similar to Experiment 1. Subjects (*N* = 20) were instructed to passively listen to the randomized stimuli while being scanned. We employed a block design in which each stimulus was presented once, with 16-s baseline silence or noise between each musical excerpt. The full scan took approximately 9 min, following which 19 subjects completed the IRI questionnaire in a quiet room. Sixteen of the participants also rated their preference for the self- and researcher-selected excerpts using a 0–100 horizontal numbered bipolar scale (“strongly dislike-strongly like”).

MRI data acquisition, preprocessing, and statistics were identical to Experiment 1. Contrasts included: (1) each category > baseline silence and noise (e.g., FL > silence/noise); (2) inter-categorical contrasts (e.g., FD > FL); (3) cross-categorical contrasts (e.g., FL > UL); (4) aggregate contrasts (e.g., L > D); and (5) interactions. As in Experiment 1, all images were thresholded at *Z* > 2.3 (*p* < 0.01), corrected for multiple comparisons across the whole brain at *p* < 0.05. IRI ratings were added as covariates in four separate analyses, and the region of activation from the task > baseline contrast was used to mask the subsequent contrasts so only regions that were also task positive by that criterion showed.

### Behavioral Results

We first examined differences between IRI scales and preference ratings in the four stimuli conditions. A test for normality of distribution (Shapiro-Wilk) resulted in the transformation of data for two IRI scales and two preference ratings (Templeton, 2011). Preference ratings for one subject were omitted as an outlier based on a criterion of ±2 SDs from the mean. As in Experiment 1, repeated-measure ANOVA revealed a significant difference between IRI subscales, *F*_(3,54)_ = 28.33, *p* < 0.0001, $\eta_{\text{p}}^{2}$ = 0.61, and as anticipated, in a *post hoc* test (Bonferroni) PD was found to differ significantly from the others (all *p* < 0.05). The four subscales registered acceptable internal consistency (*M* Cronbach’s *α* = 0.65), and were not significantly correlated after correcting for multiple comparisons (False Discovery Rate method; see Benjamini and Hochberg, 1995). To confirm main effects of the four familiarity/valence conditions on mean preference ratings, we ran another repeated-measure ANOVA, which revealed a large main effect, *F*_(3,45)_ = 152.93, *p* < 0.0001, $\eta_{\text{p}}^{2}$ = 0.91. Significance of all pairwise comparisons was confirmed at *p* < 0.0001 (Bonferroni; see Supplementary Material S2 Table 2 for descriptive statistics of Experiment 2 behavioral data).

**Figure 3:**
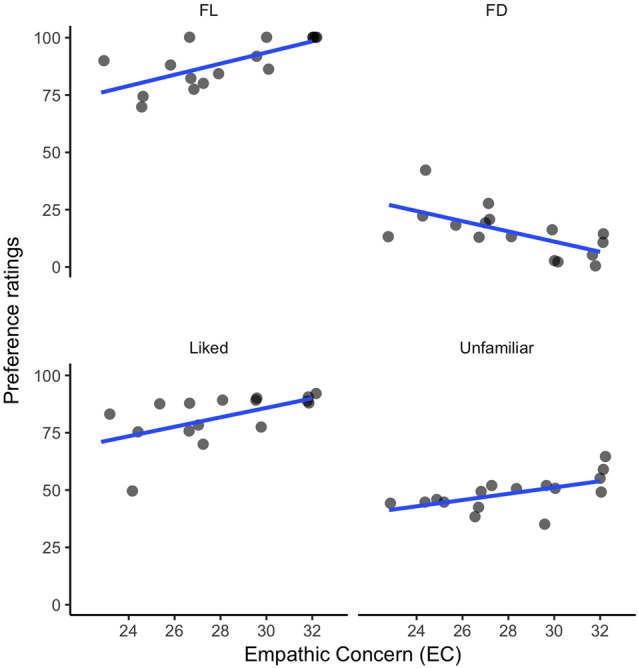
Correlations between EC and musical preference: (1) ratings for familiar liked (FL) excerpts; (2) ratings for familiar disliked (FD) music; (3) ratings for all liked excerpts (familiar and unfamiliar); and (4) ratings for all unfamiliar music (liked and disliked).

PT, FS and PD were not strongly associated with the preference ratings, but, as shown in Figure 3, EC showed strong correlations with preference for FL music, *r*_(14)_ = 0.70, *p* < 0.01; FD, *r*_(14)_ = −0.64, *p* = 0.02; liked music (i.e., both familiar and unfamiliar), *r*_(14)_ = 0.57, *p* = 0.03; and unfamiliar music (both liked and disliked), *r*_(14)_ = 0.56, *p* = 0.03, significance levels adjusted for multiple comparisons (False Discovery Rate). The correlation between empathy and liking of unfamiliar music suggests that empathic people in our sample were more likely to be affectively open-minded to new music, even excerpts they disliked.

### Imaging Results

**Figure 4:**
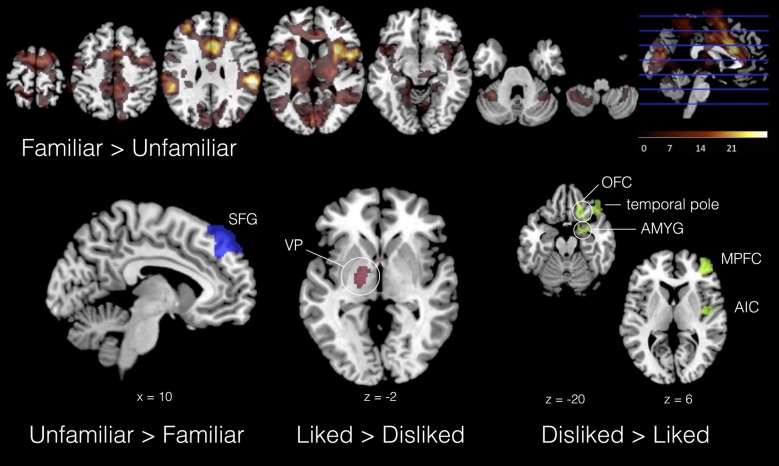
Selected results from four basic contrasts. Familiar > Unfamiliar displayed as heat map in seven axial slices; UNFAMILIAR > FAMILIAR = blue; LIKED > DISLIKED = red; DISLIKED > LIKED = green. All contrasts, *Z* > 2.3, *p* < 0.01 (cluster corrected, *p* < 0.05).

In the basic group-level analysis (no empathy covariates), we found the involvement of left ventral pallidum (VP) and thalamus in musical liking (L > D). This result is consistent with previous research on the neurophysiology of musical preference, which has broadly confirmed the role of basal ganglia reward circuitry in musical pleasure (Blood et al., 1999; Salimpoor et al., 2013). In contrast, musical disliking (D > L) was accompanied by activity in right AIC, MPFC, OFC, superior temporal gyrus (STG) and amygdala/parahippocampus. AIC has been implicated in most emotional empathy studies; it has also been found to contribute to both positive (Koelsch et al., 2006) and negative reactions to musical stimuli (Wallmark et al., 2018). Amygdala and OFC are also often involved in negative affect (Phan et al., 2002), and connectivity between these two areas is indicative of emotional regulation (Banks et al., 2007). Moreover, lateralization of affective response—left with positive, right with negative—is also consistent with other studies (Hellige, 1993). Finally, unfamiliar music was associated with enhanced activation of bilateral superior frontal gyrus (SFG) compared to familiar—possibly indicating heightened attention—while familiar music involved the contribution of a dense network of areas across the whole brain, including bilateral IPL, anterior cingulate and paraginculate, premotor cortex, SMA, medial prefrontal areas, and cerebellum (Janata et al., 2007; Pereira et al., 2011). Figure 4 displays results for these four basic contrasts (see also Supplementary Material S2 Table 3).

**Figure 5:**
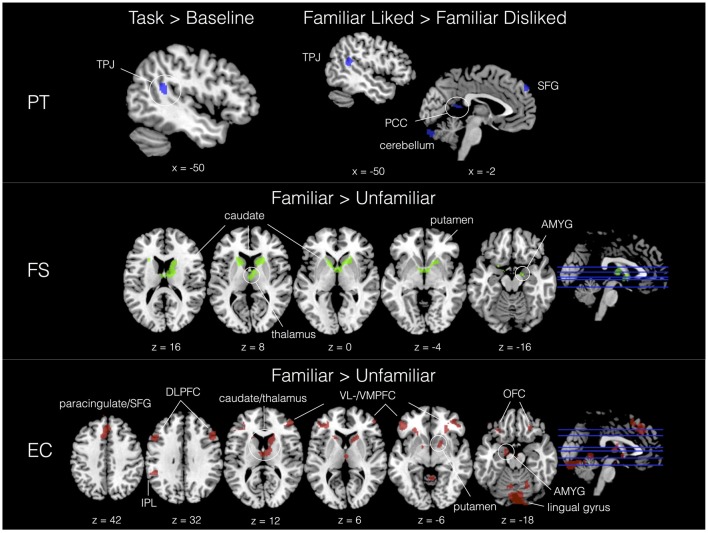
Activation sites correlating with trait empathy (IRI subscales) in selected contrasts. PT = blue, FS = green, EC = red. All contrasts, *Z* > 2.3, *p* < 0.01 (cluster corrected, *p* < 0.05).

With IRI covariates added in a second-level analysis, we found neural signatures distinctive to all subscales. As shown in Table 2, PT was associated with activity in the left TPJ in response to the task. Familiar liked music covaried with PT in cerebellum, TPJ, SFG and the posterior part of the cingulate (PCC) compared to FD music; further, in comparison with UL music, FL showed increased activation in a large region of the right PFC. A significant interaction effect between liking and familiarity was observed in the right dorsolateral PFC (DLPFC). In contrast, FS exhibited significant correlations with activity in dorsal striatum and limbic areas—including caudate, putamen, thalamus, fornix, hippocampus and amygdala—as a function of musical familiarity (F > U) and liking (FL > UL). Activations for IRI covariates in selected contrasts are shown in Figure 5.

**Table 2:** Experiment 2 results by IRI subscale.

Contrasts and regions	Cluster extent (voxels)	Maxima			
		MNI coordinates			
		*x*	*y*	*z*	*Z*
***Perspective taking (PT)***					
**Task > Baseline**					
L temporoparietal junction	92	−46	−42	16	3.13
**Familiar Liked > Familiar Disliked**					
R/L cerebellum	1350	−16	−88	−38	3.66
L temporoparietal junction	664	−48	−46	20	3.4
L superior frontal gyrus	38	−4	58	40	2.8
L posterior cingulate	32	−2	−42	12	2.99
**Familiar Liked > Unfamiliar Liked**					
R dorsolateral prefrontal	716	32	8	34	3.3
R ventrolateral prefrontal	325	46	42	−14	3.24
R medial prefrontal	40	12	46	44	2.75
**Interaction (FL–UL > FD–UD)**					
R dorsolateral prefrontal	676	52	32	28	3.36
***Fantasy (FS)***					
**Familiar > Unfamiliar**					
R/L dorsal striatum and limbic	1493	12	−2	20	4.07
**Familiar Liked > Unfamiliar Liked**					
R/L dorsal striatum and limbic	1619	18	16	−2	3.94
***Empathic concern (EC)***					
**Familiar > Unfamiliar**					
R/L cerebellum, lingual gyrus, occipital pole	2683	40	−66	−28	4.4
R/L orbitofrontal and dorsal striatum	1812	−22	28	−10	4.04
R/L dorsomedial prefrontal	875	−8	20	44	3.5
R/L inferior frontal gyrus/dorsolateral prefrontal	520	44	48	12	3.55
L amygdala	62	−14	−2	−18	2.68
L inferior parietal lobule	57	−46	−44	32	3.3
R inferior parietal lobule	35	62	−42	26	3.02
**Familiar Liked > Familiar Disliked**					
L middle temporal gyrus	339	−64	−48	6	3.27
**Familiar Liked > Unfamiliar Liked**					
R dorsolateral prefrontal	2523	42	22	36	3.48
L orbitofrontal	2042	−28	24	−12	4.08
R/L medial/ventromedial prefrontal	1290	−2	34	42	3.65
R cerebellum	1002	42	−54	−56	3.86
L cerebellum	938	−38	−66	−40	3.59
L middle temporal gyrus	293	−64	−42	−4	3.27
L inferior parietal lobule	206	−48	−44	32	3.87
R temporoparietal junction	128	58	−42	24	3.9
R ventral premotor cortex	61	40	0	62	3.46
**Familiar Disliked > Unfamiliar Disliked**					
R dorsal striatum	105	4	6	0	3.1
L orbitofrontal	56	−24	30	−6	3.12
**Unfamiliar Disliked > Unfamiliar Liked**					
R dorsolateral prefrontal	77	14	20	68	3.02
**Interaction (FL–UL > FD–UD)**					
R inferior frontal gyrus	1241	52	22	10	3.26
L orbitofrontal	262	−34	24	−16	3.22
R SMA	211	12	24	60	3.06
L temporoparietal junction	114	−58	−48	26	3.31
R dorsolateral prefrontal	105	46	8	52	3.16
***Personal distress (PD)***					
**Familiar Disliked > Unfamiliar Disliked**					
R medial prefrontal cortex	147	46	50	12	3.14

*N = 19. Significant voxels were obtained at a threshold of Z > 2.3, p < 0.01 (cluster-corrected, p < 0.05). Brain region labels for all MNI coordinates are based on the Juelich Histological Atlas (Mazziotta et al., 2001). Contrast activations ordered from top to bottom by cluster extent (most to least); brain regions and coordinates listed are derived from peak voxels within each cluster*.

EC also showed sensitivity to familiarity, which was correlated with activity in dorsomedial PFC (extending ventrally to paracingulate), IPL, DLPFC and IFG, cerebellum, visual areas (lingual gyrus and occipital pole), dorsal striatum, VMPFC, VLPFC and amygdala in the F > U contrast. This strong familiarity effect could also be seen in a number of other contrasts, including FL > FD, which showed a correlation with activity in middle temporal gyrus; FL > UL, which added activity in right posterior IFG; and the interaction between liking and familiarity. Additionally, EC revealed some rare correlations with disliked music, including the head of the caudate in the FD > UD contrast, as well as DLPFC in the UD > UL contrast. Finally, the PD scale was associated with activation in the right MFG in the FD > UD contrast. This was the only significant result for this subscale in either experiment.

## Discussion

The present study demonstrates that trait empathy is correlated with neurophysiological differences in music processing. Music has long been conceived as a social stimulus (North and Hargreaves, 2008; Livingstone and Thompson, 2009; Aucouturier and Canonne, 2017). Supporting this view, our study offers novel evidence that neural circuitry involved in trait empathy is active to a greater degree in empathic individuals during perception of both simple musical tones and full musical excerpts. Individual variances in empathy are reflected in differential recruitment of core empathy networks (Fan et al., 2011) during music listening; specifically, IRI subscales were found to correlate with activity in regions associated with both emotional (e.g., sensorimotor regions, insular and cingulate cortex) and cognitive empathy (e.g., PFC, TPJ) during passive listening tasks.

Our main hypotheses were confirmed, though with an unexpected twist regarding the two putative empathy types (at least as structured by the IRI). Both experiments seem to suggest interactions between bottom-up and top-down processes (indexed in our study by both IRI scores and activity in neural systems) in empathy-modulated music listening. This is in line with recent findings in prosocial decision making studies (Christov-Moore and Iacoboni, 2016; Christov-Moore et al., 2017a,b). Stimulus type, however, seems associated with different patterns of neural systems engagement. In Experiment 1, sensorimotor areas were more frequently modulated by trait empathy in the processing of musical timbre; conversely, in Experiment 2, cognitive areas were more frequently modulated by trait empathy in the processing of (familiar) music. Together this suggests that, contrary to our initial hypothesis for Experiment 2, modulation of neural activity by empathy was driven more by stimulus type than by empathy type; that is, the emotional empathy subscale (EC) was no more selective to emotional contagion circuitry than cognitive empathy scales (PT and FS), and vice versa (the PD scale did not reveal any significant correlations with brain activity). In what follows, we interpret these results and discuss their implications.

### Empathy-Modulated Sensorimotor Engagement in Timbre Processing

Using isolated 2-s instrument and vocal tones as stimuli, Experiment 1 found that the four IRI subscales modulated response to timbre. First, we found that cognitive perspective taking (PT) was correlated with activity in motor areas (SMA and primary motor cortex), SI and anterior cingulate (ACC). This finding is in line with numerous studies suggesting a role for ACC and SI in emotional empathy (Bufalari et al., 2007; Singer and Lamm, 2009); it also replicates a result of Gazzola et al. (2006), who reported a correlation of somatomotor activity and PT scores in an action sound listening task. Activity in these regions may suggest a sensorimotor simulation process whereby high-PT individuals imitate internally some aspect of the production of these sounds. This result could be explained in light of Cox’s (2016) “mimetic hypothesis,” according to which music is understood by way of covert or overt motor reenactments of sound-producing physical gestures. It is quite conceivable that people who are inclined to imagine themselves from others’ perspectives also tend to take up the physical actions implied by others’ musical sounds, whether a smooth and gentle voice, a growled saxophone, or any other musical sound reflecting human actions. It is intriguing, however, that PT was not implicated in the processing of positive or negative valence. One might assume that perspective takers possess a neural preference for “good” sounds: for example, one study reported activation of larynx control areas in the Rolandic operculum while subjects listened to pleasant music (but not unpleasant), suggesting subvocalization only to positively valenced music (Koelsch et al., 2006). Our results, however, indicate that PT is not selective to valence in these sensorimotor areas.

FS also revealed motor involvement (SMA) in the task > baseline contrast. Unlike PT, FS appeared to be sensitive to both positive and negative valence of timbres: we found activity in left TPJ and Broca’s area of the IFG associated with positively valenced timbres, and temporal, parietal and prefrontal activations associated with disliked timbres. TPJ is an important structure for theory of mind (Saxe and Kanwisher, 2003; Young et al., 2010). Together with Broca’s area—a well-studied language and voice-specific motor region (Watkins and Paus, 2004; Brown et al., 2008) that has been implicated in emotional empathy (Fan et al., 2011; Cheetham et al., 2014)—it is plausible to suggest that individuals who are prone to fantasizing may exhibit a greater tendency to attribute mental states to the virtual human agents responsible for making musical sounds, and that this attribution would be more pronounced for positively valenced stimuli (Warren et al., 2006).

As hypothesized, EC was correlated with activation in a range of areas previously implicated in empathy studies, including IPL, IFG and SMA, along with SI, STG, cerebellum and AIC (Iacoboni, 2009). It was also sensitive to negative valence: noisy timbres were processed with greater involvement from SMA in individuals with higher EC. EC is an “other-oriented” emotional scale measuring sympathy or compassion towards the misfortune of others (Batson, 1991; Davis, 1996). Since noisy, distorted qualities of vocal timbre are an index of generally high-arousal, negatively valenced affective states (Tsai et al., 2010), we theorize that individuals with higher trait EC exhibited greater motor attunement owing to the ecological urgency typically signaled by such sound events. In short, we usually deploy harsh vocal timbres when distressed or endangered (e.g., screaming or shouting), not during affectively positive or neutral low-arousal states, and high-empathy people are more likely to pick up on and simulate the affective motor implications of others in distress. Though our sensitivity to the human voice is especially acute (Belin et al., 2000), researchers have hypothesized that instrumental timbre can similarly function as a “superexpressive voice” via acoustic similarities to emotional vocal expression (Juslin and Västfjäll, 2008). Our result would seem to support this theory, as motor response appears to encode the combined effects of noisy tones, both vocal and instrumental.

It is also worth noting, as might be expected given the above, that noisy voice produced a unique signature of activation among high FS and EC participants relative to the normal vocal stimuli (Supplementary Material S1 Figure 1; Supplementary Material S1 Tables 1, 2): FS modulated processing of the noisy voice in SII and IPL, while EC was selective to noisy vocal sounds in the SMA and primary motor cortex. This result appears to be at odds with other studies of vocal affect sensitivity that report motor-mimetic selectivity for pleasant vocalizations (Warren et al., 2006; Wallmark et al., 2018). It is likely that individual variances in empathy (plus other mediating factors) predispose listeners to differing orientations towards others’ affective vocalizations, with empathic listeners more likely to “catch” the motor-affective implications of aversive sounds than low-empathy people, who might only respond to sounds they find pleasant while tuning out negatively valenced vocalizations. Cox (2016) theorizes that music can afford listeners an “invitation” for motor engagement, which they may choose to accept or decline. Seen from this perspective, it is likely that individual differences in empathy play an important role in determining how we choose to respond to music’s motor invitations.

Regarding motor engagement, across IRI subscales it is apparent that SMA is the most prominent sensorimotor area involved in empathy-modulated processing of timbre. SMA is a frequently reported yet undertheorized part of the core empathy network (Fan et al., 2011); it has also been implicated in internally generated movement and coordination of action sequences (Nguyen et al., 2014), and has been shown in a single-neuron study to possess mirror properties (Mukamel et al., 2010). Most relevant to the present study, moreover, SMA contributes to the vividness of auditory imagery, including imagery for timbre (Halpern et al., 2004; Lima et al., 2015). Halpern et al. (2004) attributed SMA activity in an auditory imagery task in part to subvocalization of timbral attributes, and the present study would seem to partially corroborate this explanation. We interpret this result as a possible instance of sensorimotor integration: SMA activity could reflect a basic propensity to link sounds with their associated actions, which are internally mirrored while listening. In accordance with this view, we would argue that people do not just passively listen to different qualities of musical timbre—they enact some of the underlying physical determinants of sound production, whether through subvocalization (Halpern et al., 2004), biography specific act-sound associations (Bangert et al., 2006; Margulis et al., 2009), or other theorized mechanisms of audio-motor coupling (Cox, 2016).

To summarize, sensorimotor areas have been implicated in many previous studies of emotional empathy, including IFG and IPL (Carr et al., 2003; Shamay-Tsoory, 2010); “pain circuit” areas in AIC and ACC (Singer et al., 2004; Shamay-Tsoory, 2010); and somatomotor regions (i.e., pre/primary motor cortices and SI/SII; Carr et al., 2003; Gazzola et al., 2006; Pfeifer et al., 2008). Interestingly, these precise regions dominated results of the Experiment 1 timbre listening task. This is true, moreover, for both emotional and cognitive scales: PT and FS, though often implicated in cognitive tasks (Banissy et al., 2012), were found in this experiment to modulate SMA, SI, primary motor cortex, IPL, AIC and IFG, well-documented motor-affective areas. We theorize that the contextual impoverishment and short duration of the timbre listening task (2-s isolated tones) may have largely precluded any genuine perspective taking or fantasizing from occurring—it is much harder to put oneself in the “shoes” of an single isolated voice or instrument, of course, than it is an affectively rich piece of actual music. However, even in the absence of conscious cognitive empathizing, which presumably would have been reflected in engagement of the cognitive empathy system, individuals with high trait PT and FS still showed selective activations of sensorimotor and affective relay circuits typically associated with emotional empathy. This could be interpreted to suggest that the two “routes” to empathy are not dissociated in music listening: although conscious PT in response to abbreviated auditory cues is unlikely, people who frequently imagine themselves in the positions of others also exhibit a tendency toward motor resonance in this basic listening task, even when musical context is missing.

### Prefrontal and Reward Activation During Music Listening

Experiment 2 used 16-s excerpts of self- and experimenter-selected music to explore the effect of dispositional empathy on the processing of music in four conditions, familiar liked (FL), familiar disliked (FD), unfamiliar liked (UL), and unfamiliar disliked (UD). Participants consisted of individuals who reported regularly experiencing intense emotional reactions while listening to music. Musical liking is associated at the group level (i.e., no IRI covariates) with left basal ganglia reward areas, and disliking with activity in right AIC, primary auditory cortex and prefrontal areas (OFC and VLPFC). Musical familiarity is associated with activation across a broad region of the cortex, subcortical areas, and cerebellum, including IPL, premotor cortex and the core empathy network (Fan et al., 2011), while unfamiliarity recruits only the SFG. This robust familiarity effect is even more acute among high-empathy listeners: after adding empathy covariates to our analysis, there were no regions that demonstrated an affect-specific response after controlling for familiarity. This result is consistent with the literature in showing a large neurophysiological effect of familiarity on musical liking (Pereira et al., 2011); it appears that trait empathy, as well, modulates responses to familiar music to a greater degree than unfamiliar music.

Contrary to expectations, activation in regions primarily associated with emotional empathy (e.g., sensorimotor areas, ACC, AIC) was not a major component in empathy-modulated music processing. Instead, the most prominent activation sites for PT and EC scales were prefrontal, including medial, lateral, and orbital portions of the cortex, as well as TPJ. These regions are involved in executive control, regulation of emotions, mentalizing, contextual appraisal, and “enactment imagination” (Goldman, 2006), and have figured prominently in many studies on the neurophysiology of cognitive empathy (Decety and Grèzes, 2006; Frith and Frith, 2006). Additionally, FS and EC results were characterized by dorsal striatum when participants listened to familiar music. This basal ganglia structure has been frequently reported in empathy studies but not often discussed (Fan et al., 2011); it has also long been associated with musical pleasure (Blood et al., 1999; Salimpoor et al., 2011, 2013). Replicating this association, our results suggest that empathic people experience a higher degree of reward and motivation when listening to familiar music compared to lower-empathy people.

PT was associated with left TPJ in the task > baseline contrast. Activation of this region among perspective-takers is consistent with studies implicating TPJ in theory of mind (Saxe and Kanwisher, 2003) and the merging of self and other (Lawrence et al., 2006). The TPJ was joined by posterior cingulate, cerebellum and superior prefrontal areas when listening to familiar liked music (FL > FD), the former two of which were also identified in a study on the neural bases of perspective taking (Jackson et al., 2006). Interestingly, these results differ substantially from the PT correlations in Experiment 1, which were entirely sensorimotor. In the context of isolated musical sounds, PT results were interpreted as a reflection of covert imitation (or, enactive perspective taking): in contrast, however, it appears here that PT may reflect a more cognitively mediated, mental form of perspective taking, which conceivably extends beyond action-perception coupling of musicians’ affective motor cues to encompass contextual appraisal, assessments of the affective intent embodied in the music, and other executive functions.

In contrast to the prominent TPJ and prefrontal activation associated with PT, FS results revealed activation of dorsal striatum (caudate and putamen) and limbic areas (thalamus, hippocampus and amygdala). Activation of reward and emotion centers may suggest that fantasizers also tend to exhibit heightened positive emotional reactions to familiar music. Indeed, we found a moderate correlation between FS and preference ratings for familiar liked music, *r*_(14)_ = 0.52, which may tentatively corroborate this claim. Moreover, structural brain studies have found that FS is associated with increased gray matter volume in hippocampus (Cheetham et al., 2014), an important memory area, perhaps also indicating enhanced encoding of familiar liked music among fantasizers.

The contrast in activation between the two IRI cognitive empathy scales (PT and FS) is notable, and may be attributed to the different aspects of empathy they were designed to assess. PT taps the tendency to imagine oneself in other people’s shoes, whereas FS captures the tendency to imagine oneself from the perspective of fictional characters (Davis, 1980, 1996). With this distinction in mind, one could surmise that the two scales also tap different views regarding the ontology of the musical agent: in this reading, people with high trait PT are more likely to take music as a social stimulus, i.e., as if it was a real or virtual human presence (with theory of mind, goals, beliefs), while high FS listeners are more likely to hear it as “fictional” from a social perspective, i.e., as a rewarding sensory stimulus with an attenuated grip on actual social cognition. Further research is called for to explore possible explanations for the differences in cognitive scales as reflected in music listening.

Turning finally to emotional empathy, we found that EC recruits prefrontal, reward and sensorimotor-affective areas in music listening, and is likewise quite sensitive to familiarity. In the Familiar > Unfamiliar contrast, we found activation of cerebellum, IPL, DLPFC, IFG, DMPFC, amygdala, anterior paracingulate, dorsal striatum, OFC and lingual gyrus, and a variation on this general pattern for the Familiar liked > Unfamiliar liked and interaction contrasts. Activation of bilateral IPL and IFG is consistent with mirror accounts of empathy (Shamay-Tsoory, 2010). Furthermore, the ACC, paracingulate, and areas that extend dorsally (SMA, DMPFC) have been proposed as the core of the empathy network (Fan et al., 2011): our result would seem to extend support for the primacy of this region using an experimental task that is not explicitly social in the manner of most empathy studies. Lastly, DLPFC is an important executive control area in cognitive empathy (Christov-Moore and Iacoboni, 2016), and has been implicated in emotional regulation (Ochsner et al., 2004; Quirk and Beer, 2006). Activation of this region may reflect top-down control over affective responses to familiar music, both in terms of up-regulation to liked music and down-regulation to disliked (or possibly up-regulation to negative stimuli, as open-minded empathic listeners try to “see something positive” in the disliked music). In further research, connectivity analysis between DLPFC and limbic/reward areas may help to specify the neurophysiological mechanisms underlying empathy-modulated emotional regulation during music listening.

In addition to motor, cingulate and prefrontal activity, we found the recruitment of emotion and reward processing areas as a function of EC and musical familiarity: dorsal striatum (the whole extent of the caudate nucleus, plus thalamus) may reflect increased pleasure in response to familiar music among empathic listeners. It is not surprising that the reward system would show preferential activation to familiar music (Pereira et al., 2011), as confirmed in the basic group Liked > Disliked contrast. Prevalence of basal ganglia for both EC and FS suggests that trait empathy may effectively sensitize people to the music they already know. This even appears to be the case for *disliked* music, which showed dorsal striatum activation (along with OFC) in the Familiar disliked > Unfamiliar disliked contrast. This could be interpreted to indicate that empathic people may experience heightened musical pleasure even when listening to the music they self-select as “hating,” provided it is familiar. By way of contrast, no striatum activation was found for any of the unfamiliar music conditions. In concert with limbic circuitry, then, it is apparent that musical familiarity recruits a broad region of the affect-reward system in high EC listeners.

Activation of inferior parts of the lingual gyrus and occipital lobe was another novel finding, and may also be linked to musical affect. These areas are associated with visual processing, including perception and recognition of familiar sights and emotional facial expressions (Kitada et al., 2010), as well as visual imagery (Kosslyn et al., 2001). It is reasonable to think that empathic listeners may be more prone to visual imagery while listening to familiar music. Visual responses are an important mechanism of musical affect more generally, and are a fairly reliable index of musical engagement and attention (Juslin and Västfjäll, 2008): if high-EC people are more susceptible to musical affect, as suggested by our results, they may also show a greater tendency towards visual imagery in music listening. To be clear, we did not explicitly operationalize visual imagery in this study: in the future, it would be interesting to follow up on this result by comparing visual imagery and music listening tasks using the EC scale as a covariate.

The behavioral data resonate in interesting and sometimes contradictory ways with these imaging findings. We found that EC is strongly associated with preference for liked music and unfamiliar music, and negative responses to familiar disliked music. Results suggest that high-EC people are more responsive to the affective components of music, as reflected in polarity of preference responses. EC was also associated with open-mindedness to new music (i.e., higher ratings for unfamiliar music), though imaging results for this contrast did not reach significance, and might appear to be contradicted by the clear familiarity effect discussed previously. We must be cautious in the interpretation of these findings owing to the small sample size, but this resonance between behavioral and imaging evidence is nonetheless suggestive in demonstrating a role for EC in affective responsiveness to familiar music. This conclusion is broadly consistent with previous behavioral studies (Egermann and McAdams, 2013), especially regarding pleasurable responses to sad music (Garrido and Schubert, 2011; Vuoskoski et al., 2012; Eerola et al., 2016).

In sum, the present results provide complementary neural evidence that involvement of prefrontal areas and limbic/basal ganglia in music listening covaries with individual trait differences in empathy, with sensorimotor engagement playing a smaller role. How do we account for the prominence of cognitive, prefrontal areas in music listening but not musical timbre in isolation? It must be noted that a broad swath of the emotional empathy system was involved in the basic task > baseline contrast (used to mask all IRI covariates): in other words, it is clear that music in aggregate is processed with some level of sensorimotor, paralimbic, and limbic involvement, regardless the empathy level of the listeners or the valence/familiarity of the music (Zatorre et al., 2007). However, our results seem to suggest that empathic people tend to be more attuned to the attribution of human agency and affective intention in the musical signal, as indicated by preferential engagement of cognitive empathy networks including PFC (MPF and DLPFC) and TPJ (Banissy et al., 2012), as well as reward areas. In other words, what seems to best characterize the high-empathy response to musical stimuli is the tendency to take an extra cognitive step towards identification with some agentive quality of the music, over and above the work of emotional contagion mechanisms alone. Thus while patterns of neural resonance consistent with emotional contagion appear to be common to most experiences of music—and were also found among high-empathy participants in Experiment 1—activation of prefrontal cognitive empathy systems for the PT and EC scales may indicate the tendency of empathic listeners to try to “get into the heads” of composers, performers, and/or the virtual persona of the music (Levinson, 2006). This top-down process is effortful, imaginative, and self-aware, in contrast to the automatic and pre-reflective mechanisms undergirding emotional contagion. Accordingly, as suggested by Schubert (2017), the involvement of cognitive systems may not strictly speaking be required for affective musical response, which can largely be accounted for by emotional contagion circuitry alone. A number of studies have shown that mental imagery may be supported by sensorimotor and affective components without the contribution of prefrontal areas (Decety and Grèzes, 2006; Ogino et al., 2007). Nevertheless, they could betoken a more social cognitive mode of listening, a deliberative attempt on the part of listeners to project themselves into the lived experience of the musical agent. This imaginative projection is more intense, understandably, for music that empathic people already know, and also appears to interact with musical preference.

### General Implications

The present study has a number of implications for social and affective neuroscience, music psychology, and musicology. For neuroscientific empathy research, we demonstrate the involvement of the core empathy network and mirror neuron system outside of tasks that are explicitly social cognitive. Most studies use transparently social experimental tasks and stimuli to assess neural correlates of state and trait empathy; for example, viewing pictures or videos of other people (for review, see Singer and Lamm, 2009). This study demonstrates that musical sound, which is perhaps not an obvious social stimulus, can elicit neural responses consistent with theories of empathy. By doing so, this study highlights the potential value of operationalizing artistic and aesthetic experience as a window into social cognitive and affective processing, a perspective that is arguably the historical progenitor of contemporary empathy research (Lipps, 1907).

For music psychology, this research has at least three main implications. First, this study demonstrates that trait empathy may modulate the neurophysiology of music listening. Although there is mounting behavioral and psychophysiological evidence pointing to this conclusion (Miu and Vuoskoski, 2017), this is the first study to investigate the effects of empathy on the musical brain. Second, this study confirms and extends empirical claims that music cognition is inextricably linked to social cognition (Huron, 2001; North and Hargreaves, 2008). Following Schubert’s (2017) common coding model, our results suggest that aspects of affective music processing can be viewed as a specialized subprocess of general social-affective perception and cognition. This may begin to explain the neural bases for how music can function as a “virtual social agent” (Leman, 2007). Third, in demonstrating neural differences in music processing as a function of empathy, we highlight the possible significance of looking at other trait features when assessing the functional neural correlates of musical tasks and stimuli. Many neurophysiological music studies take only a few trait features into account in sampling procedures and analysis, most notably sex, age, and musical training: the latter has been well explored (e.g., Alluri et al., 2017), but other factors—such as personality factors and mood—are not frequently addressed. Individual differences in music processing may relate to dispositional characteristics that can be captured by psychosocial questionnaires, indirect observational techniques, or other methods. Exploring the role of such trait variables in musical behaviors and brain processing could provide a more detailed and granular account of music cognition.

Finally, these results enrich the humanistic study of music in providing a plausible psychobiological account for the social valence of musical experience observed in diverse cultural and historical settings. As music theorist Clifton (1983) claims, “the ‘other’ need not be a person: it can be music.” In a very rough sense, this study provides empirical support for this statement: areas implicated in trait empathy and social cognition also appear to be involved in music processing, and to a significantly greater degree for individuals with high trait empathy. If music can function something like a virtual “other,” then it might be capable of altering listeners’ views of *real* others, thus enabling it to play an ethically complex mediating role in the social discourse of music (Rahaim, 2017). Indeed, musicologists have historically documented moments of tense cultural encounter wherein music played an instrumental role in helping one group to realize the other’s shared humanity (Cruz, 1999). Recent research would seem to provide behavioral ballast for this view: using an implicit association task, Vuoskoski et al. (2016) showed that listening to the music of another culture could positively modulate attitudes towards members of that culture among empathic listeners. Though we do not in this study explicitly address whether music can alter empathic brain circuits, it is suggestive that certain attitudes toward musical sound may have behavioral and neural bases in individual differences in trait empathy.

### Limitations

A few important limitations must be considered in interpreting these results. First, this study was correlational: no causative links can thus be determined in the relationship between music and trait empathy. In the future, it would be interesting to use an empathy priming paradigm (Miu and Balteş, 2012) in an fMRI context to compare neurophysiological correlates of trait empathy with primed “state” empathy in music listening; this could provide a powerful method for disentangling possible differences in processing between dispositional attributes of empathy and contextual factors (e.g., socially conditioned attitudes about a performer, mood when listening). As a corollary to the above, moreover, this study does not address whether our results are specific to music listening: perhaps high-empathy people utilize more of these areas when performing other non-musical yet not explicitly social tasks as well (e.g., viewing abstract art). Additionally, we do not explore whether there could be other mediating trait factors in music processing besides empathy and sex: personality and temperament, for instance, have been shown to modulate responses to music (Rentfrow and Gosling, 2003). Finally, this study will need to be replicated with a larger sample size, and with participants who do not self-select based on strong emotional reactions to music, in order to strengthen the statistical power and generalizability of the results (Yarkoni, 2009).

## Conclusion

In two experiments using fMRI, this article demonstrates that trait empathy modulates music processing. Replicating previous findings in the social neuroscience literature, isolated musical timbres are related to sensorimotor and paralimbic activation; in actual music listening, however, empathy is primarily associated with activity in prefrontal and reward areas. Empathic participants were found to be particularly sensitive to abrasive, “noisy” qualities of musical timbre, showing preferential activation of the SMA, possibly reflecting heightened motor-mimetic susceptibility to sounds signaling high-arousal, low-valence affective states. In the music listening task, empathic subjects demonstrated enhanced responsiveness to familiar music, with musical preference playing a mediating role. Taken together, these results confirm and extend recent research on the link between music and empathy, and may help bring us closer to understanding the social cognitive basis for music perception and cognition.

## Author Contributions

ZW and MI conceptualized and designed the experiments and wrote the article. ZW performed the experiments. CD and ZW analyzed the data and visualized the data. MI and CD consulted on the article.

## Conflict of Interest Statement

The authors declare that the research was conducted in the absence of any commercial or financial relationships that could be construed as a potential conflict of interest.
