# Supplementary material for: Neurophysiological Effects of Trait Empathy in Music Listening
Source: Front Behav Neurosci. 2018 Apr 6;12:66. doi: 10.3389/fnbeh.2018.00066 (PMC5897436; doi:10.3389/fnbeh.2018.00066)
Supplement: Supplementary file 1 [file Data_Sheet_1.DOCX]

Supplementary Material

Neurophysiological effects of trait empathy in music listening

Zachary Wallmark*, Choi Deblieck, Marco Iacoboni

*** Correspondence:** Zachary Wallmark: zwallmark@smu.edu

# Supplementary Data, Tables, Figures, and Stimuli

All supplementary data—including NIFTI files, tables, figure, and stimuli referenced in the manuscript text—are included in the attached “data sheet” zip directory.
