# Supplementary material for: Neurophysiological Effects of Trait Empathy in Music Listening
Source: Front Behav Neurosci. 2018 Apr 6;12:66. doi: 10.3389/fnbeh.2018.00066 (PMC5897436; doi:10.3389/fnbeh.2018.00066)
Supplement: Supplementary file 2 [file Data_Sheet_2.ZIP › Supplementary materials/S1 table 1.docx]

**S1 Table 1: Experiment 1 summary of behavioral data**

| **IRI subscales** | ***M*** | ***SD*** | **95% CI** |
| --- | --- | --- | --- |
| Perspective taking | 27.56 | 3.68 | [24.73, 30.38] |
| Fantasy | 27.11 | 5.80 | [22.65, 31.57] |
| Empathic concern | 29.22 | 3.49 | [26.54, 31.91] |
| Personal distress | 18.56 | 5.41 | [14.40, 22.71] |
| **Preference ratings** |  |  |  |
| Normal timbres (1) | 55.19 | 9.18 | [48.14, 62.25] |
| Noisy timbres (2 + 3) | 43.76 | 14 | [33, 54.5] |
| Affective polarity (normal–noisy) | 11.43 | 11.61 | [2.51, 20.35] |

*N =* 14. The highest IRI subscale score possible is 35 (7 questions x 5 points each).
