# Supplementary material for: Neurophysiological Effects of Trait Empathy in Music Listening
Source: Front Behav Neurosci. 2018 Apr 6;12:66. doi: 10.3389/fnbeh.2018.00066 (PMC5897436; doi:10.3389/fnbeh.2018.00066)
Supplement: Supplementary file 2 [file Data_Sheet_2.ZIP › Supplementary materials/S1 table 2.docx]

**S1 Table 2: Voice-specific results by IRI scale**

| **Scales, contrasts, and regions** | **Cluster extent (voxels)** | **Maxima**  **MNI coordinates** | | | |
| --- | --- | --- | --- | --- | --- |
|  |  | *x* | *y* | *z* | *Z* |
| ***Fantasy* (FS)** | | | | | |
| **Noisy voice > Normal voice** |  |  |  |  |  |
| R secondary somatosensory | 81 | 58 | -20 | 22 | 3.36 |
| L inferior parietal lobule | 17 | -40 | -64 | 30 | 3.36 |
| ***Empathic concern* (EC)** | | | | | |
| **Noisy voice > Normal voice** |  |  |  |  |  |
| R/L SMA | 431 | 8 | -6 | 74 | 3.74 |
| R/L primary motor cortex | 56 | 8 | -24 | 62 | 2.97 |

*N* = 14. Significant voxels were obtained at a threshold of Z > 2.3, *p* < .01 (cluster-corrected, *p* < .05). Brain region labels for all MNI coordinates are based on the Juelich Histological Atlas. Contrast activations ordered from top to bottom by cluster extent (most to least); brain regions and coordinates listed are derived from peak voxels within each cluster.
