# Supplementary material for: Neurophysiological Effects of Trait Empathy in Music Listening
Source: Front Behav Neurosci. 2018 Apr 6;12:66. doi: 10.3389/fnbeh.2018.00066 (PMC5897436; doi:10.3389/fnbeh.2018.00066)
Supplement: Supplementary file 2 [file Data_Sheet_2.ZIP › Supplementary materials/S2 table 2.docx]

**S2 Table 2: Experiment 2 summary of behavioral data**

| **IRI subscales** | ***M*** | ***SD*** | **95% CI** |
| --- | --- | --- | --- |
| Perspective taking | 27.26 | 4.32 | [25.18, 29.34] |
| Fantasy | 26.32 | 5.52 | [23.66, 28.98] |
| Empathic concern | 28.84 | 3.34 | [27.23, 30.46] |
| Personal distress | 18.24 | 4.91 | [15.87, 20.60] |
| **Preference ratings** |  |  |  |
| Familiar like (FL) | 93.13 | 11.94 | [86.76, 99.48] |
| Familiar dislike (FD) | 15.14 | 10.54 | [9.52, 20.76] |
| Unfamiliar like (UL) | 71.06 | 11.58 | [64.89, 77.23] |
| Unfamiliar dislike (UD) | 26.22 | 10.13 | [20.82, 31.62] |
| Liking affective polarity (L–D) | 61.41 | 17.59 | [52.04, 70.79] |
| Familiarity affective polarity (F–U) | 5.49 | 8.04 | [1.21, 9.78] |

IRI *N =* 19, preference *N =* 16. The highest IRI subscale score possible is 35 (7 questions x 5 points each).
