# Supplementary material for: Neurophysiological Effects of Trait Empathy in Music Listening
Source: Front Behav Neurosci. 2018 Apr 6;12:66. doi: 10.3389/fnbeh.2018.00066 (PMC5897436; doi:10.3389/fnbeh.2018.00066)
Supplement: Supplementary file 2 [file Data_Sheet_2.ZIP › Supplementary materials/S2 Table 3.docx]

**S2 Table 3: Results of basic contrasts (no IRI covariates) in four contrasts**

| **Regions** | **Cluster extent (voxels)** | **Maxima**  **MNI coordinates** | | | |
| --- | --- | --- | --- | --- | --- |
|  |  | *x* | *y* | *z* | *Z* |
| **Liked > Disliked** | | | | | |
| L dorsal striatum | 597 | -24 | 2 | 16 | 13.4 |
| **Disliked > Liked** | | | | | |
| R ventrolateral prefrontal | 486 | 44 | 46 | -10 | 13.46 |
| R superior temporal gyrus | 102 | 46 | -12 | 4 | 15.39 |
| R orbitofrontal cortex | 91 | 26 | 14 | -20 | 12.21 |
| R anterior insula | 72 | 48 | -14 | -8 | 14.37 |
| **Familiar > Unfamiliar** | | | | | |
| L inferior parietal lobule | 14860 | -60 | -40 | 30 | 26.44 |
| L cerebellum | 1672 | -28 | -58 | -54 | 21.35 |
| R premotor cortex | 639 | 42 | 2 | 58 | 18.44 |
| L medial prefrontal | 492 | -30 | 46 | 26 | 23.21 |
| R cerebellum | 294 | 30 | -60 | -56 | 16.53 |
| R medial prefrontal | 47 | 28 | 52 | 24 | 11.16 |
| **Unfamiliar > Familiar** | | | | | |
| L/R superior frontal gyrus | 454 | -4 | 40 | 42 | 12.44 |

*N* = 20. Significant voxels were obtained at a threshold of Z > 2.3, *p* < .01 (cluster-corrected, *p* < .05). Brain region labels for all MNI coordinates are based on the Juelich Histological Atlas. Contrast activations ordered from top to bottom by cluster extent (most to least); brain regions and coordinates listed are derived from peak voxels within each cluster.
