# Supplementary figures and images for: Neurophysiological Effects of Trait Empathy in Music Listening
Source: Front Behav Neurosci. 2018 Apr 6;12:66. doi: 10.3389/fnbeh.2018.00066 (PMC5897436; doi:10.3389/fnbeh.2018.00066)

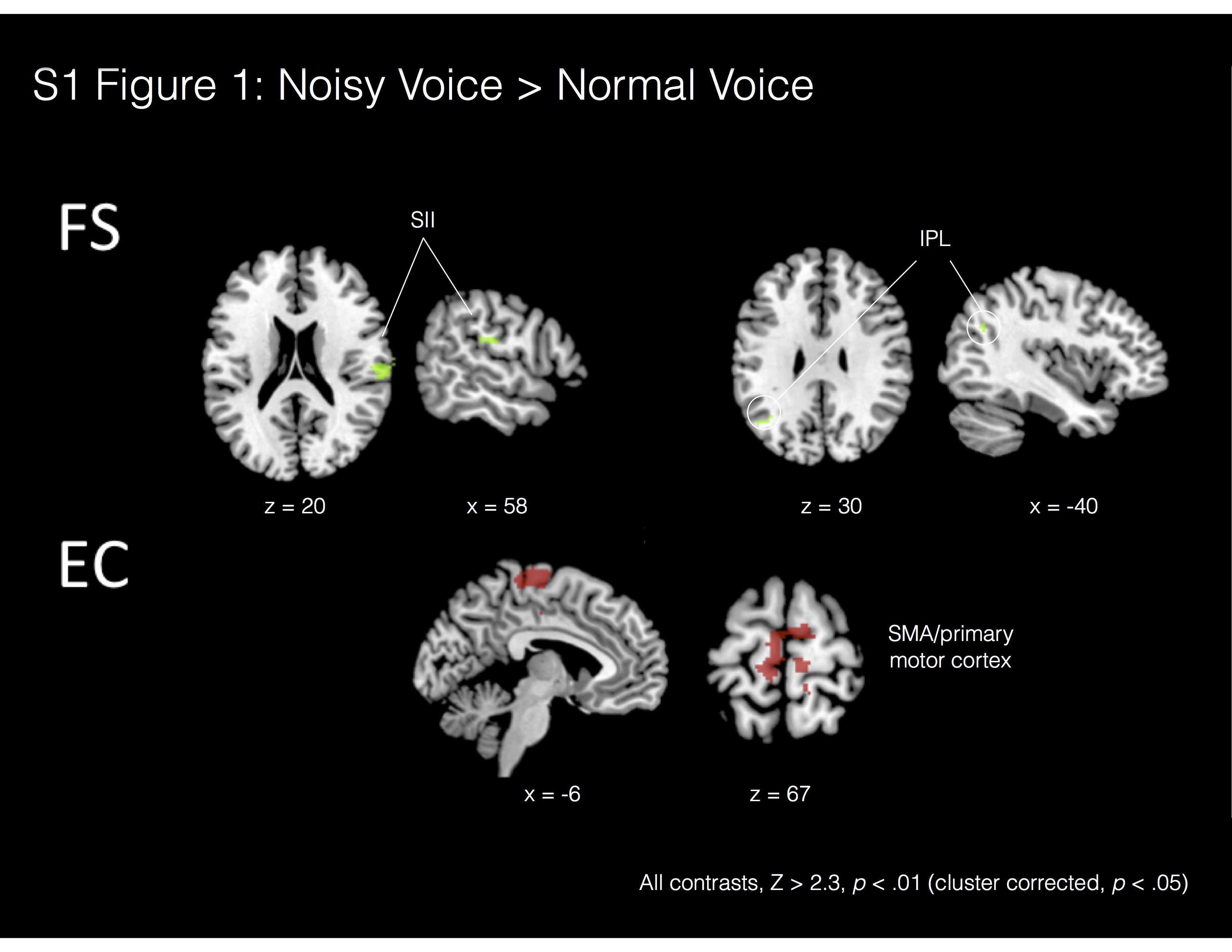

Supplement: Supplementary file 2 [file Data_Sheet_2.ZIP › Supplementary materials/S1 figure 1.jpg]
